# A Computer Simulation Study of Thermal and Mechanical Properties of Poly(Ionic Liquid)s

**DOI:** 10.3390/membranes12050450

**Published:** 2022-04-21

**Authors:** Youngseon Shim, Munbo Shim, Dae Sin Kim

**Affiliations:** Innovation Center, Samsung Electronics Co., Ltd., 130 Samsungjeonja-ro, Hwaseong-si 16678, Gyeonggi-do, Korea; munbo.shim@samsung.com (M.S.); daesin.kim@samsung.com (D.S.K.)

**Keywords:** poly(ionic liquid)s, glass transition temperature, mechanical modulus, molecular dynamics simulation

## Abstract

Thermal and mechanical properties of poly(ionic liquid)s (PILs), an epoxidized ionic liquid-amine network, are studied via molecular dynamics simulations. The poly(ionic liquid)s are designed with two different ionic liquid monomers, 3-[2-(Oxiran-2-yl)ethyl]-1-{4-[(2-oxiran-2-yl)ethoxy]phenyl}imidazolium (EIM2) and 1-{4-[2-(Oxiran-2-yl)ethyl]phenyl}-3-{4-[2-(oxiran-2-yl)ethoxy]benzyl}imidazolium (EIM1), each of which is networked with tris(2-aminoethyl)amine, paired with different anions, bis(trifluoromethanesulfonyl)imide (TFSI^−^) and chloride (Cl^−^). We investigate how ionic liquid monomers with high ionic strength affect structures of the cross-linked polymer networks and their thermomechanical properties such as glass transition temperature (T_g_) and elastic moduli, varying the degree of cross-linking. Strong electrostatic interactions between the cationic polymer backbone and anions build up their strong structures of which the strength depends on their molecular structures and anion size. As the anion size decreases from TFSI^−^ to Cl^−^, both T_g_ and elastic moduli of the PIL increase due to stronger electrostatic interactions present between their ionic moieties, making it favorable for the PIL to organize with stronger bindings. Compared to the EIM2 monomer, the EIM1 monomers and TFSI^−^ ions generate a PIL with higher T_g_ and elastic moduli. This attributes to the less flexible structure of the EIM1 monomer for the chain rotation, in which steric hindrance by ring moieties in the EIM1-based PIL enhances their structural rigidity. The π-π stacking structures between the rings are found to increase in EIM1-based PIL compared to the EIM2-based one, which becomes stronger with smaller Cl^−^ ion rather than TFSI^−^. The effect of the degree of the cross-linking on thermal and mechanical properties is also examined. As the degree of cross-linking decreases from 100% to 60%, T_g_ also decreases by a factor of 10–20%, where the difference among the given PILs becomes decreased with a lower degree of cross-linking. Both the Young’s (E) and shear (G) moduli of all the PILs decrease with degree of cross-linking, which the reduction is more significant for the PIL generated with EIM2 monomers. Transport properties of anions in PILs are also studied. Anions are almost immobilized globally with very small structural fluctuations, in which Cl^−^ presents lower diffusivity by a factor of ~2 compared to TFSI^−^ due to their stronger binding to the cationic polymer backbone.

## 1. Introduction

Supported ionic liquid phases have been extensively emerging as a new class of functional materials, where ionic liquids with low volatility, chemical and thermal stability, and ionic conductivity are immobilized on polymers or inorganic supports [1,2,3,4,5,6,7]. A polymer-supported ionic liquid has important implications for advanced materials in the area of catalysis as well as energy applications. It was found that supported ionic liquid phases yield similar or even better catalytic performance in terms of activity, selectivity, and stability than conventional organic solvents [8,9,10]. Poly(ionic liquid)s (PILs) attract more attention as alternative polymer electrolytes in various applications such as in electrochemical, energy storage, and conversion devices [11,12,13,14,15,16,17]. Despite their high thermal stability and wide electrochemical window as electrolytes in lithium-based batteries, their commercial market is restricted because of low ionic conductivities at ambient temperature. An electrolyte for lithium batteries requires an ionic conductivity above $10^{-1}$ S/m, while polymer electrolytes appear to reach an upper limit below $10^{-2}$ S/m at room temperature. The performance of PILs can be further enhanced by adding ILs in the solvent to increase the mobility of PIL chains [18,19,20,21]. The ionic conductivity of PILs should be improved in a future direction towards the design of mobile ionic moieties with more flexibility in a robust macromolecular structure, and practical synthetic strategies.

Thermosetting polymers enable the high-temperature applications with strong mechanical properties due to their three-dimensional network structure by cross-linking. To take advantage of ionic properties for thermosetting polymers, new ionic liquid monomers were designed and polymerized to generate eco-friendly PILs with novel physical properties for potential electrochemical and biomedical applications [22,23,24,25,26,27,28]. Considerable efforts have already been exerted on the polymerization using ionic liquid monomers with high ionic conductivity, thermal and electrochemical stability, low vapor pressure, and nonflammability, targeting the sustainable polymer networks with improved properties [29,30,31,32,33,34]. In recent years, there have been several research developments on the polymerization using ionic liquid monomers with antibacterial and mechanical functions. Epoxy-amine networks designed from imidazolium ionic liquid-based monomers were found to play a crucial role in the hydrophobic behavior for antibacterial functions due to the chemical structure of ionic liquids [35,36]. Livi et al., investigated the reaction and kinetics between the imidazolium ionic liquid monomer and poly(oxypropylene diamine) using differential scanning calorimetry analyses (DSC), Fourier-transform infrared (FTIR) and “in situ” NMR spectroscopy and their physical properties such as the thermal stability, thermomechanical behavior, and ionic conductivity ($2.4\times 10^{-3}$ S/m at 373 K) were also demonstrated using transmission electronic microscopy (TEM), thermogravimetric analysis (TGA), dynamical mechanical analysis (DMA), and dielectric spectroscopy [36]. Polymer networks, polymerized between the bis(imidazolium) ionic liquid and isophorone diamine, were reported by Radchenko et al., in which their hydrophobic behavior, thermal and mechanical properties were examined using TGA, DMA, and DSC [37]. Dzienia et al., demonstrated the cure kinetics of DGEBA using imidazolium-based ionic liquid curing agents, in which the presence of the salicylate anion results in a highly cross-linked polymerization compared to chloride anion [38]. Radchenko et al., reported the synthesis of PIL networks based on cycloaliphatic epoxidized ionic liquids with thermomechanical and potential shape memory behaviors [39,40].

The potential application of PILs as functional materials in energy storage and electrochemical devices is promising as the new types of ionic liquids are being developed with the advances in polymer chemistry. The synthesis of PILs has been extensively studied in recent decades, but fundamental understanding of the mechanisms underlying structure–property relations in PILs is still far from the requirements of practical energy storage and electrochemical devices. The key issue we examine in this article is how thermal and mechanical properties of PILs are influenced by their microscopic structures. To address this issue, we investigate how different ionic liquid monomers affect glass transition temperatures and elastic moduli of their polymer networks, varying the degree of cross-linking. The effects of anions on the structures and properties of the PILs are also examined. Finally, we also demonstrate transport dynamics of the anions in the PILs. The outline of this paper is as follows: In Section 2, we give a brief description of the models and methods employed in this study. Equilibrium structural properties and dynamics of the PILs are analyzed and compared with other recent experimental studies in Section 3. Concluding remarks are offered in Section 4.

## 2. Models and Methods

**Figure 1:**
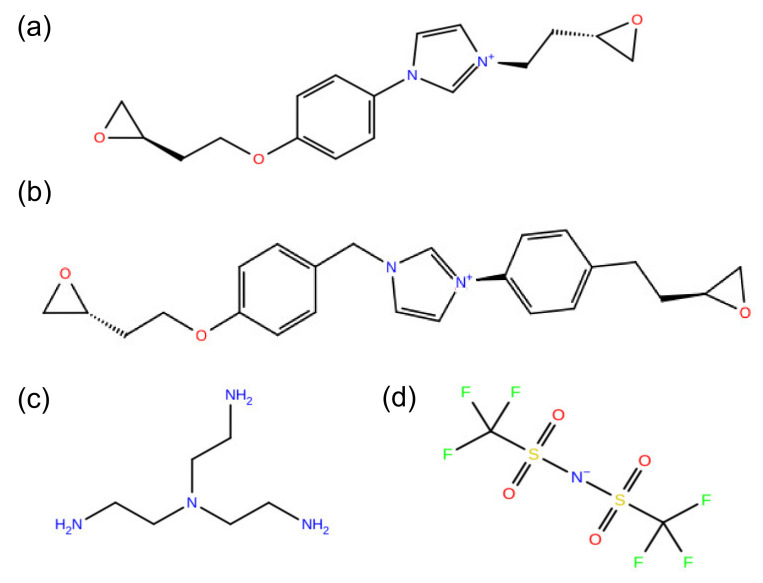
Molecular structures of cationic (**a**) EIM2 and (**b**) EIM1 monomers, (**c**) tris(2-aminoethyl)amine (TAEA) curing agent, and (**d**) bis(trifluoromethanesulfonyl)imide (TFSI^−^) anion.

**Figure 2:**
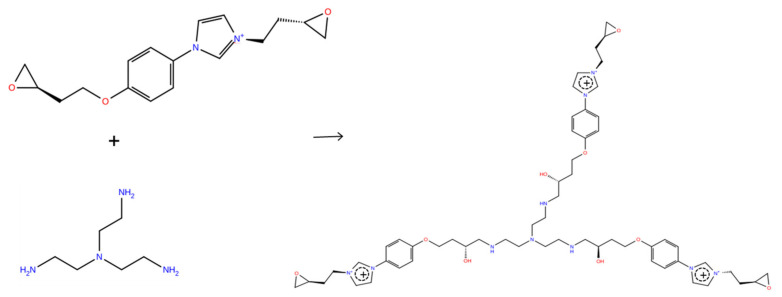
A representation of the cross-linking processes and their networked structures, obtained by reactions of cationic EIM2 monomers and tris(2-aminoethyl)amine (TAEA) curing agent. The network structure propagates by cross-linking processes in the three-dimensional space, where the stoichiometry for EIM2 monomers and TAEA is set to be 3:2 in the system, paired with anions to neutralize the total system.

The model PIL systems composed of bis(epoxidized) imidazolium monomers networked with tris(2-aminoethyl)amine (TAEA), paired with either bis(trifluoromethanesulfonyl)imide (TFSI^−^) or chloride (Cl^−^), were studied using molecular dynamics (MD) simulations. We considered two different imidazolium-based cationic monomers, 3-[2-(Oxiran-2-yl)ethyl]-1-{4-[(2-oxiran-2-yl)ethoxy]phenyl}imidazolium (EIM2) and 1-{4-[2-(Oxiran-2-yl)ethyl]phenyl}-3-{4-[2-(oxiran-2-yl)ethoxy]benzyl}imidazolium (EIM1), where the cationic monomers are paired with either TFSI^−^ or Cl^−^ anions to be electronically neutral. All molecular structures of cationic monomers, TFSI^−^, and TAEA curing agent are described in Figure 1. All simulations were performed using Schrodinger’s Materials Science Suite program [41]. The OPLS3e force field parameters [42,43] were used to describe the PILs. Each system was initially set by randomly packing the 800 molecules in a simulation box with a stoichiometry of 3:2 for ionic liquid monomers and TAEA. We utilized a cross-linking simulation based on distance-based reaction criteria to mimic curing processes of PILs, employing the TAEA curing agent. The ratio of reaction rates, epoxy-primary amine reactions k_1_ and epoxy-secondary amine reactions k_2_, was set as 1. The cross-linked topologies after curing processes and the final properties could be significantly changed by the ratio of reaction rates. However, it was confirmed that no significant difference between the T_g_ predicted for k_2_/k_1_ = 1 and k_2_/k_1_ = 0.01 was observed for the systems composed of diglycidyl-ether of bisphenol F (DGEBF) and 4,4′-diaminodiphenyl sulphone (44DDS) [44]. One of the cross-linked reaction structures between cationic monomers and TAEA is displayed in Figure 2. To examine the effect of degree of cross-linking φ for PILs, the cross-linking iterations were φ =60−100% in 10% increments. One of the cross-linked structures between cationic monomers and TAEA is displayed in Figure 2. As for φ =100%, 300 cationic monomers are completely cross-linked together as a single cationic polymer backbone by 200 TAEA curing molecules, paired with 300 anions to neutralize the system.

For the PILs cross-linked at each degree of cross-linking φ, MD simulations for 20 ns were performed using NPT ensemble at 700 K and 1 atm and then five different configurations with a time interval of 1 ns were collected to make five annealing cycles. To evaluate T_g_ of the PILs at each degree of cross-linking φ, the annealing cycles of each system were conducted over a temperature range from 700 to 200 K, in 20 K decrements. At each temperature, the densities were determined using NPT ensemble over the last 2 ns trajectories of the total 20 ns trajectories, averaged over the five samples. The long-range electrostatic interactions were computed via the Ewald method, resulting in essentially no truncation of these interactions. The trajectories were integrated via RESPA algorithm using a time step of 1 fs [45]. The Nosé–Hoover thermostat [46] and Martyna–Tobias–Klein barostat [47] methods were used.

The hyperbolic-regression model of the density ρ averaged over the five cooling cycles, as a function of temperature T [48],

(1) $$\rho \left(T\right)=\rho_{0}-a\left(T-T_{0}\right)-bH_{0}\left(T-T_{0},c\right)$$

(2) $$H_{0}\left(T-T_{0},c\right)=\frac{1}{2}\left(T-T_{0}\right)+\sqrt{\frac{{\left(T-T_{0}\right)}^{2}}{4}+e^{c}}$$

is utilized to evaluate the glass transition temperature T_g_, where *T*_0_, $\rho_{0}$, a, b, and c are determined to fit $\rho \left(T\right)$. The constants -a and –a-b are the slopes of the asymptotic low and high temperature regimes, and c smoothens the discontinuity in the slope close to *T* = *T*_0_. The T_g_ is estimated by the intersection of low and high temperature asymptotes of the hyperbola.

Equilibration simulations for 1 μs at each cross-linking φ were performed in the canonical ensemble at 300 K, followed by 20 ns production trajectories from which ensemble averages were computed to calculate the structure and dynamic properties. Mechanical properties of PILs were examined with elastic constants to characterize their stiffness. The linear stress–strain relation in the limit of infinitesimal deformation is given by

(3) $$\sigma_{ij}=C_{ijkl}\epsilon_{kl}$$

(4) $$\sigma_{ij}=\frac{1}{V}\overset{N}{\underset{\alpha =1}{\sum }}[-m_{\alpha }v_{i}^{\alpha }\;v_{j}^{\alpha }+\overset{N}{\underset{\beta <\alpha }{\sum }}(r_{i}^{\beta }-r_{i}^{\alpha })f_{i}^{\alpha \beta }]$$

where $\sigma_{ij}$ and $\epsilon_{kl}$ represent the elements of the symmetric stress and strain tensor, and $C_{ijkl}$ are the elements of the fourth rank elasticity tensor. The instantaneous stress $\sigma_{ij}$ is computed based on the virial theorem, where indices α, β refer to atoms and $f_{i}^{\alpha }$, $v_{i}^{\alpha }$, and $r_{i}^{\alpha }$ are the *i-*th force, velocity, and position component of atom α. *V* is the volume of the atomic system and N is the number of atoms in the system. MD simulations at each degree of cross-linking were performed using NVT ensemble at 300 K and 1 atm by applying a constant strain rate of 2 × 10^6^/s under uniaxial and biaxial tensions. The stress and strain data were averaged over each direction and last 20% of 2 ns trajectories and elastic moduli were calculated using the tensile stress–strain curve up to 1.6%.

## 3. Results and Discussion

**Figure 3:**
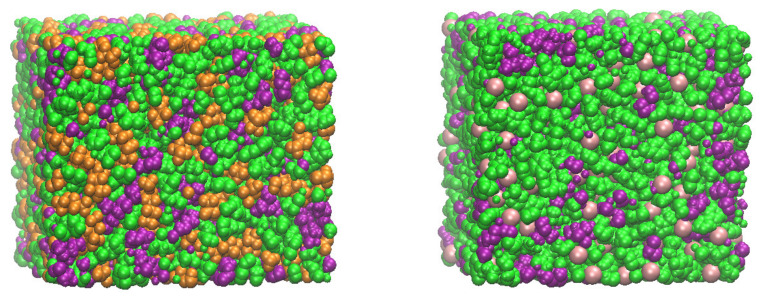
Snapshots of the PILs generated with EIM2 cationic monomers and different anions, TFSI^−^ (left-hand side) and Cl^−^ (right-hand side), obtained at 300 K. Van der Waals representations of the molecules are employed, where green, purple, orange, and pink symbols represent the atoms of the EIM2, TAEA, TFSI^−^, and Cl^−^.

**Figure 4:**
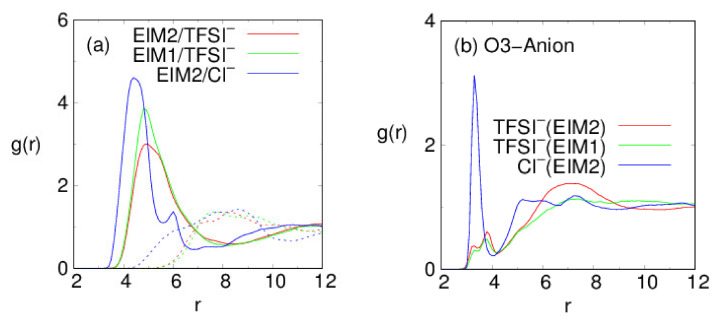
Radial distributions g(r) of (**a**) the cationic EIM2/EIM1 (in solid lines) and TFSI^−^/Cl^−^ ions (in dashed lines) around the TFSI^−^/Cl^−^ ions in the PILs, and g(r) of (**b**) TFSI^−^/Cl^−^ ions around the ether oxygens (O3) of EIM2/EIM1, obtained 300 K, where r (in units of Å) is a distance between the center-of-mass of the anions and (**a**) nitrogen (imidazolium)/(**b**) oxygen (ether) atoms of the EIM2/EIM1.

We first demonstrate the molecular structure of the PILs. The model PIL systems composed of bis(epoxidized) imidazolium monomers networked with tris(2-aminoethyl)amine (TAEA), paired with either bis(trifluoromethanesulfonyl)imide (TFSI-) or chloride (Cl-), are presented in snapshots of Figure 3. To examine the intermolecular structure between the cationic polymer backbone and anions, their radial distribution functions g(r) are displayed in Figure 4a, where r is a distance between the center-of-mass of the anions and nitrogen atoms of the imidazolium ring in the PIL backbone. It is remarkable that anions in the three PILs make a strong structure (in solid lines) over r =4∼6 Å, followed by the minimum structure around r =7∼9 Å, regardless of φ. To be specific, TFSI^−^ and Cl^−^ build up their maximum distribution at r =4.4 and 4.8 Å, respectively, from nitrogens of the EIM2-based backbone. This is ascribed to the fact that smaller Cl^−^ anions more strongly interact with cationic imidazolium rings in a shorter interionic distance, compared to TFSI^−^. It is also found that TFSI^−^ anions organize stronger structures at the EIM1-based backbone rather than the EIM2-based one. A decrease in the interionic distance in Figure 4a gives rise to the increase in the electrostatic interaction strength. Compared to cationic imidazolium-anion structures, TFSI^−^ and Cl^−^ ions exhibit broad anion–anion distributions (in dashed lines) over r =7∼10 Å and r =5∼10 Å, respectively. We note that cationic imidazoliums and anions are distributed in an alternative layered manner, in similarity with room temperature ionic liquids [49]. As a result, the difference in ion sizes and their molecular structures determine the strength of their electrostatic interactions. On the other hand, the backbones of all three PILs are found to connect by hydrogen bonds to ether oxygens of the EMI2/EIM1, where a hydrogen bond is defined to exist if the distance between the two heavy atoms (O--O) is less than 2.8 Å and the angle of H--O--O is less than 30°. The amount of the ether oxygens forming hydrogen bonds decreases in order of PIL system generated with EIM2/TFSI^−^ (88–91%), EIM1/TFSI^−^ (76–80%), and EIM2/Cl^−^(28–35%), respectively, over φ =60–90%. We note that the hydrogen bonding characteristic has dramatically reduced for EIM2/Cl^−^, in which Cl^−^ ions have a tendency to make a strong peak with oxygen atoms replacing the hydrogen bonds, displayed in Figure 4b.

**Figure 5:**
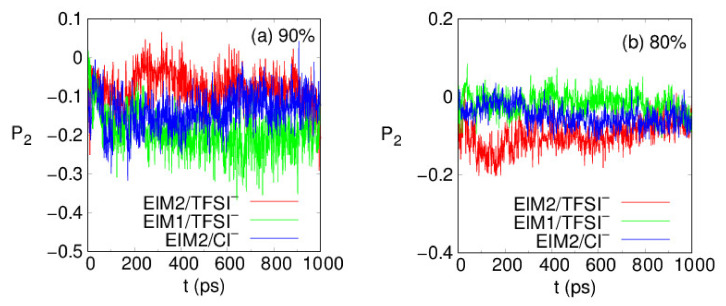
Order parameters $P_{2}(\cos\theta \left(t\right))$ of the PILs at $\left(a\right)\;\varphi \;=90\;\mathrm{and}\;\left(b\right)\;80\%$ at 300 K, where $P_{2}$ is the second order Legendre polynomial and $\cos\theta \left(t\right)$ is the angle between the ring orientation and its *z*-axis of the simulation box at time t.

To gain more insight into the PIL structure, the orientational order and ring stacks of the PIL were scrutinized to associate their structural rigidity. The order parameters $<P_{2}(\cos\theta \left(t\right))>$ for PILs are analyzed, where $\langle \cdots \rangle$ denotes an equilibrium average, $P_{2}$ is the second order Legendre polynomial, and $\cos\theta \left(t\right)$ is the angle between the ring plane and its *z*-axis of the simulation box at time t. We notice that the ring orientation becomes isotropic $P_{2}∼0$ with smaller orientational fluctuations as the φ decreases in Figure 5. The magnitude of $P_{2}$ is slightly increased for the EIM1-based PIL of which the monomer has one more ring than EIM2. As for the EIM1-based PIL, $P_{2}∼-0.2$ indicates that on average the rings there are tilted from the *z*-axis by about 65°. The π−π stacking structure between the rings such as imidazolium and benzene is also counted when the distance between the center-of-mass of the rings is within 4.4Å and the angle between the two ring planes is less than 30°. The total number of π−π stacking structures increases for the EIM1-based PIL of which the monomer has one more ring than EIM2. As for the same EIM2, Cl^−^ anions generate a slightly greater number of π−π stacking structures. We note that smaller Cl^−^ make distributions around the cationic backbone with slightly less disturbance of the π−π stacking orientations between the rings of the backbone compared to TFSI^−^. The ratio of the π−π stacking structures is denoted as $P_{\pi -\pi \;}$, averaged over all the rings. For three PILs, $P_{\pi -\pi \;}$ increases in order of EIM2/TFSI^−^ (53–57%), EIM1/TFSI^−^ (54–60%), and EIM2/Cl^−^ (56–61%) over φ =60–90%, where $P_{\pi -\pi \;}$has a tendency to reduce with increase in φ. The rotational motions of the ring moieties at the polymer backbone are hindered due to their high dihedral free energy barriers, increasing steric hindrance due to their limited structural reorganization in the local free volume [50]. The structural orientations and rigidity arising from ring moieties would affect thermal and mechanical properties.

**Figure 6:**
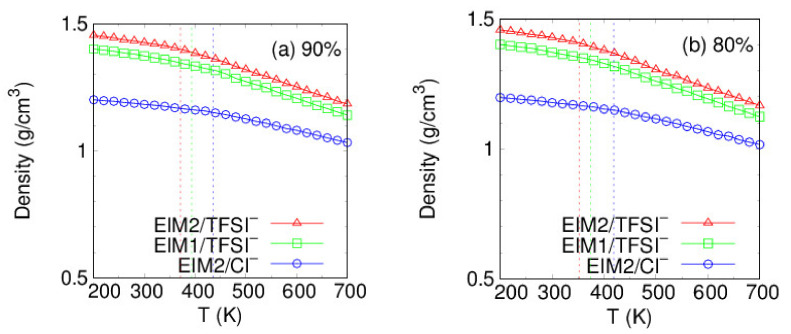
Temperature-dependent density profiles of the PILs at $\left(a\right)\;\varphi \;=90\;\mathrm{and}\;\left(b\right)\;80\%$.

**Figure 7:**
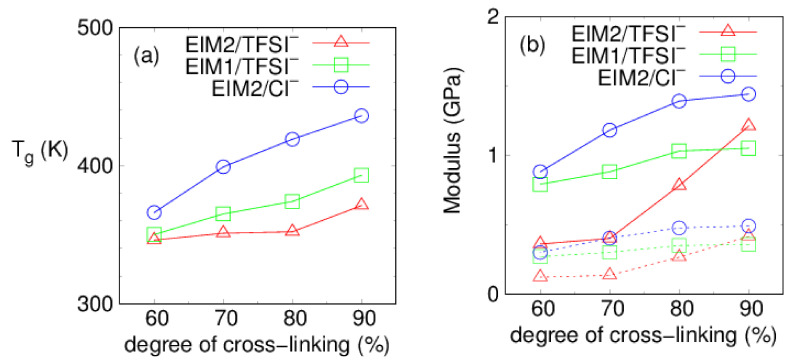
Glass transition temperature (**a**) T_g_ and (**b**) elastic moduli, obtained at 300 K. Young’s and shear moduli are displayed in solid and dotted lines, respectively. Error bars are within symbol sizes.

To measure the glass transition temperature T_g_, density profiles of the PILs obtained from the annealing cycles were used over φ =60–90% and the values of T_g_ were determined by Equation (1). Temperature-dependent density behaviors at φ =80 and 90% are demonstrated in Figure 6. The total density of PILs decreases with increase in the electrostatic interactions between the cationic imidazoliums and anions, in order of system generated with EIM2/TFSI^−^, EIM1/TFSI^−^, and EIM2/Cl^−^. The MD results of glass transition temperature T_g_ at each degree of cross-linking φ are exhibited in Figure 7a and Table 1. Our MD results agree that the glass transition temperature increases with increasing the degree of the cross-linking [51]. The predictions of T_g_ from MD simulations are usually higher than experimental values because of the discrepancy in their cooling rates. MD simulations typically cool the system by more than 10 K per nanosecond, while cooling rates in experiments are set to be of the order of 10 K per minute. The predictions are commonly corrected to subtract approximately 3 K for each order of magnitude difference in cooling rates according to the well-established Williams–Landel–Ferry (WLF) equation [52]. As for the EIM2- and EIM1-based PILs paired with TFSI^−^, the corrected T_g_ of the PILs are estimated to be 317~342 K and 321~364 K, respectively, over the degree of cross-linking 60–90%. As the anion size decreases from TFSI^−^ to Cl^−^ for the EIM2, T_g_ increases by 20 K at φ =60% to 65 K at φ =90% due to the stronger electrostatic interactions present between their ionic moieties, making it favorable for the PIL to organize with stronger binding. It was also found that T_g_ for the EIM1 monomer is higher by 22 K at φ =90% compared to the EIM2 monomer and the difference is reduced with decrease in the φ. This attributes to the fact that dihedral motions of ring moieties in the EIM1-based PIL are hindered to enhance the structural rigidity.

McDanel et al., studied PILs composed of bis(epoxidized) ionic liquid monomer and TFSI^−^, and networked with TAEA, using differential scanning calorimetry (DSC), where T_g_ = 311 and 282 K with the epoxy conversion rate of 67 and 80% were measured at different monomer stoichiometric ratios [34]. Their monomer would be more flexible without any aromatic ring compared to our EIM2 and EIM1, resulting in lowering T_g_. Livi et al., studied PILs composed of the same IL monomers and anions as ours, but networked with different curing agent Jeffamine D-230, using DSC and DMA [35]. It was found to be T_g_ = 328 and 340 K for PILs generated with EIM2 and EIM1 monomers, respectively, where the epoxy conversion rates were higher than 90%. The trend in T_g_ between EIM2- and EIM1-based PILs is in agreement with our predicted value above, that is, EIM1 monomers tend to build up stiffer PILs with higher T_g_. We note that the glass transition temperatures of an epoxy network can be hugely changed more than 140 K by just using different curing agents [53,54]. This can explain larger T_g_ values obtained in our simulations. It is notable that T_g_ of PILs could be lower compared to the conventional epoxy networks based on diglycidylether bisphenol A (DGEBA) cured with various amines, where triethylenetetramine (TETA), isophorone diamine (IPD), and diethyltoluene diamine (DETDA) were known to make the epoxy networks with high T_g_ = ~400 K, ~440 K, and 440~480 K, respectively [54,55,56]. Whereas, a bisphenol A group composed of two benzene rings connected by a dimethylmethane in DGEBA increase steric hindrance due to their very limited chain rotations in the local free volume and thus T_g_, ionic liquids monomers such as EIM1 and EIM2 are found to decrease T_g_ of the polymer networks.

MD results of the elastic moduli are presented in Figure 7b and Table 1. Young’s modulus E range is 1.1~1.4 GPa for our three PILs at φ =90%, where the values decrease with decrease in φ in our PILs. As we increase φ up to 100%, E is dramatically increased to be 3.2~3.5 GPs for our PILs paired with TFSI^−^. Our predicted E values can be larger than those measured experimentally due to the discrepancy between the strain rates used in the simulations and experiments. The conventional epoxy networks based on diglycidylether bisphenol A (DGEBA) cured with various amines were known to have Young’s modulus of E = 2.4~2.7 GPa [54,55,56]. The polymer copolymerized between the bisimidazolium ionic liquid monomers and IPD was reported to show E = 1.7 GPa and T_g_ = 328~338 K at the conversion rate of more than 95%, using DSC and DMA [37]. We notice that our PILs paired with TFSI^−^ have relatively high Young’s modulus of 1.1–3.5 GPa at φ =90–100%, despite lower T_g_ compared to DEGBA. Analogous to the glass transition temperature, elastic properties of the PILs, both Young’s modulus (E), and shear modulus (G), are found to increase with increasing the degree of cross-linking [51].

**Figure 8:**
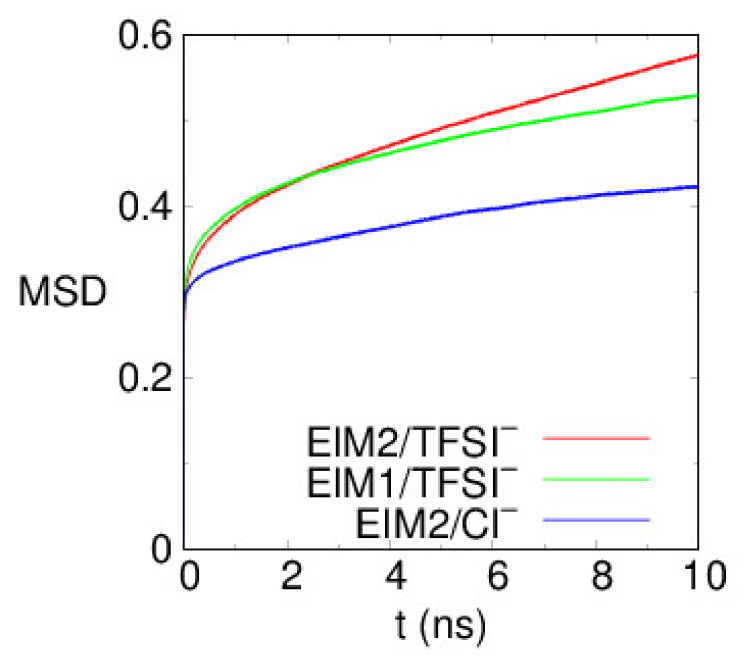
Mean square displacements (MSDs) of the center-of-mas of TFSI^−^ and Cl^−^ ions in the PILs, obtained at 300 K. The units of MSDs are Å^2^.

We take into account translational motions of the anions in the PILs to examine the ion mobility for potential applications to electrolytes. In Figure 8, mean square displacements (MSDs) of TFSI- and Cl- ions in the PILs at φ =90% are displayed. In the long time limit of normal diffusion, they are assumed to increase linearly with time, and a self-diffusion coefficient can be calculated from their slope. We note that translational motions of the anions in the PILs are much slower with diffusion coefficients of about 5×10^−5^~10^−4^ fick (1 fick = 10^−9^ m^2^/s) at room temperature, whereas the corresponding value in the ionic liquids, alkyl-imidazolium TFSI^−^, is three orders of magnitude larger at about 10^−2^~10^−1^ fick [57,58]. The translational motions of Cl^−^ ions significantly decelerate compared to those of TFSI- ions, attributed to stronger binding to the cationic segments of the PILs in Figure 3 left. TFSI^−^ presents higher diffusivity by factor of ~2 than smaller Cl^−^ due to their weaker binding to the cationic polymer backbone. Analogous to our MD results, recent computational studies have been studied to examine the mechanism of ion transport. The anion diffusivity in poly(1-butyl-3-vinylimidazolium-tetrafluoroborate) was also found to be three orders of magnitude lower than that in ionic liquids, where the hopping of an anion trapped in cages organized by cationic segments of the chain determines their diffusivity [59]. Mogurampelly et al., reported that the ion mobility is strongly correlated to the average lifetimes of the ion associations in poly(ionic liquid)s [60]. Luo et al., also reported that the anion mobility is correlated to both the structural relaxation times and the average lifetime of ion associations [61]. They also observed that a small halide anion has a significantly lower diffusivity with dynamic heterogeneity, ascribed to the stronger bind of the anions with the polymerized cation. Our MD results imply that ion transport of the PILs is governed by intermolecular structures in the PILs and their binding strength between the inter-ions. Recently, a cross-linked epoxy-based solid-state polymer electrolyte combined with ionic liquids, tetraethylene glycol dimethyl ether, and Li salt, was reported to exhibit a high ionic conductivity of ~10^−1^ S/m, depending on the Li salt concentration and associated polymerization-induced phase separation [62,63]. It was pointed out that the molecular interactions according to the Li salt concentration cause different phase separation and morphologies, affecting the activation energy for the ion conduction [63].

## 4. Conclusions

We investigated thermal and mechanical properties of PILs generated with different ionic liquid monomers using molecular dynamics computer simulations. Effects of microscopic molecular structures on glass transition temperatures and elastic moduli of the polymer networks were examined, varying the degree of cross-linking. Strong electrostatic interactions between the cationic polymer backbone and anions build up their strong structures of which the strength depends on their molecular structures and anion size. As electrostatic interactions present between their ionic moieties increase, the PIL shows strongly binding structures. As either anion size increases from Cl^−^ to TFSI^−^ or cationic monomer becomes flexible, both T_g_ and elastic moduli of their PIL are reduced. It was found that thermal and mechanical properties, T_g_, tensile and shear modulus increase with degree of cross-linking in the PILs. Anion transport in the PILs was also examined through their translational dynamics. Anions strongly binding to cationic moieties are almost immobilized globally with limited structural fluctuations, where Cl^−^ presents lower diffusivity by factor of ~2 than TFSI^−^. Tunable combinations of their cationic moieties and anions allows to develop ionic liquids with improved ionic conductivity in a future direction towards the design of mobile ionic moieties including more flexible spacers, robust macromolecular structures, and practical synthetic strategies. Another strategy to enhance the ionic conductivity is the co-addition of solvent plasticizers into the polymer electrolytes [18,19,20,21], resulting in a more flexible one with lower glass transition temperature and mechanical strength. To improve the ionic mobility without any loss of elastic modulus, phase-separated microstructures of the cross-linked polymer networks could be also designed [62,63]. Influence of the microscopic structures at the molecular level on thermomechanical properties should be understood for applications to practical energy storage and electrochemical devices.

## Figures and Tables

**Table 1:** Glass transition temperatures Tg (in K) and Young’s modulus E (in GP) for poly(ionic liquid)s according to the degree of cross-linking φ(%). The poly(ionic liquid)s consist of bis(epoxidized) imidazolium monomers (EIM2/EIM1) networked with TAEA, paired with either bis(trifluoromethanesulfonyl)imide (TFSI^−^) or chloride (Cl^−^) anions. The elastic properties are obtained at 300 K.

φ(%)	T_g_ (K)			E (GPa)		
	EIM2/TFSI^−^	EIM2/Cl^−^	EIM1/TFSI^−^	EIM2/TFSI^−^	EIM2/Cl^−^	EIM1/TFSI^−^
90	371	436	393	1.21	1.44	1.05
80	352	419	374	0.78	1.39	1.03
70	351	399	365	0.40	1.18	0.88
60	346	366	350	0.36	0.88	0.79

## Data Availability

Not applicable.
